# Genetic Diversity and Population Structure of Two Tomato Species from the Galapagos Islands

**DOI:** 10.3389/fpls.2017.00138

**Published:** 2017-02-15

**Authors:** Yveline Pailles, Shwen Ho, Inês S. Pires, Mark Tester, Sónia Negrão, Sandra M. Schmöckel

**Affiliations:** ^1^Biological and Environmental Sciences and Engineering Division, King Abdullah University of Science and TechnologyThuwal, Saudi Arabia; ^2^Genomics of Plants Stress Unit, Instituto de Tecnologia Química e Biológica António Xavier, Universidade Nova de Lisboa and Instituto de Biologia Experimental e TecnológicaOeiras, Portugal; ^3^Department of Biology and Center for Genomics and Systems Biology, New York UniversityNew York, NY, USA

**Keywords:** genotyping-by-sequencing, *Solanum cheesmaniae*, *Solanum galapagense*, genetic diversity, biogeography, tomato, wild relatives, Galapagos Islands

## Abstract

Endemic flora of the Galapagos Islands has adapted to thrive in harsh environmental conditions. The wild tomato species from the Galapagos Islands, *Solanum cheesmaniae* and *S. galapagense*, are tolerant to various stresses, and can be crossed with cultivated tomato. However, information about genetic diversity and relationships within and between populations is necessary to use these resources efficiently in plant breeding. In this study, we analyzed 3,974 polymorphic SNP markers, obtained through the genotyping-by-sequencing technique, DArTseq, to elucidate the genetic diversity and population structure of 67 accessions of Galapagos tomatoes (compared to two *S. lycopersicum* varieties and one *S. pimpinellifolium* accession). Two clustering methods, Principal Component Analysis and STRUCTURE, showed clear distinction between the two species and a subdivision in the *S. cheesmaniae* group corresponding to geographical origin and age of the islands. High genetic variation among the accessions within each species was suggested by the AMOVA. High diversity in the *S. cheesmaniae* group and its correlation with the islands of origin were also suggested. This indicates a possible influence of the movement of the islands, from west to east, on the gene flow. Additionally, the absence of *S. galapagense* populations in the eastern islands points to the species divergence occurring after the eastern islands became isolated. Based on these results, it can be concluded that the population structure of the Galapagos tomatoes collection partially explains the evolutionary history of both species, knowledge that facilitates exploitation of their genetic potential for the identification of novel alleles contributing to stress tolerance.

## Introduction

Biodiversity in the Galapagos Islands has inspired theories of adaptation and evolution, and increased our understanding of processes of population divergence and speciation (Darwin, 1859). The volcanic origin and tectonic activity of the Galapagos Islands makes them a unique site for studying the impacts of isolation and environment on diversification. The islands were formed at a volcanic hotspot in the Nazca Plate, which is moving east at approximately 59 km per million years (Geist et al., 2014). The age of the islands can be estimated by their current distance from the hotspot: the western islands are millions of years younger than the eastern ones (Geist et al., 2014). The eastward movement of the Nazca Plate causes its subduction beneath the South American plate, isolating previously interconnected islands and causing their eventual disappearance from east to west (Christie et al., 1992). The isolation of the islands and constantly changing environmental conditions have allowed the adaptation and divergence of many species, differing morphologically and genetically from one island to the other (Romagosa et al., 2013).

**FIGURE 1:**
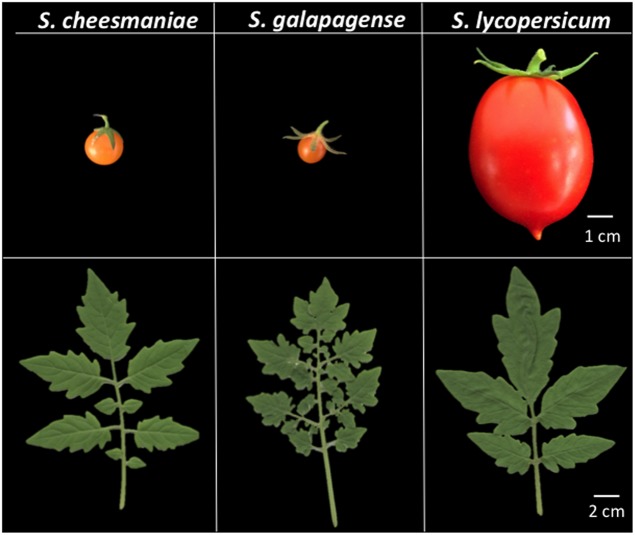
**Morphological differences between *Solanum cheesmaniae*, *S. galapagense*, and *S. lycopersicum.* Upper panels present representative images of mature fruit.** Lower panels show representative images of leaf architecture. *S. cheesmaniae* has one to two-pinnately compound leaves, short calyx and yellow to orange fruit color, while *S. galapagense* is distinguished by its three to four-pinnately compound leaves, dense pubescence, wide corolla segment, large accrescent calyx, thin pericarp, and dark orange-red fruit color (Rick, 1983). Accessions shown are *S. cheesmaniae* (LA0421), *S. galapagense* (LA0317), and *S. lycopersicum* (LA4345*).*

The vascular flora of the Galapagos Islands includes around 550 species, of which approximately 200 are endemic (Lawesson et al., 1987). Of particular interest are two wild tomato species, *Solanum cheesmaniae* (formerly *Lycopersicon cheesmanii)* and *S. galapagense* (formerly *L. cheesmanii* forma *minor)*, collectively termed “Galapagos tomatoes” (**Figure 1**). Both species of Galapagos tomatoes were first considered as one. However, based on clear morphological differences and molecular evidence from an allozyme analysis, Darwin et al. (2003) described them as two different species. The adaptation of these wild germplasms to different environments, such as arid or saline soils, makes them a potential valuable source of genetic variation in terms of stress tolerance genes, which could be transferred into commercial varieties by introgression breeding (Zamir, 2001; McCouch, 2004). However, to efficiently utilize this wild germplasm resource, it is necessary to understand the population structure and genetic variation (Lv et al., 2012). This will assist breeders in selecting germplasm that are more diverse and prevent the less efficient crossing of accessions that are very closely related. It also makes screening of the wild germplasm more efficient by enabling the selection of highly diverse accessions.

Next-generation sequencing technologies, such as geno-typing-by-sequencing (GBS) and “Diversity Arrays Technology” (DArTseq), now allow genome-wide fingerprinting without prior genome sequence information (James et al., 2008). The GBS approach can be more informative than predesigned single nucleotide polymorphism (SNP) arrays when applied to wild germplasm because it is unbiased and includes information on rare alleles (Wenzl et al., 2004, 2007; James et al., 2008). What makes DArTseq different from other GBS methods is their complexity reduction approach, targeted to select genome fractions with coding regions (Cruz et al., 2013). The restriction enzymes used in DArTseq for complexity reduction separate low copy sequences from the repetitive regions of the genome (Tinker et al., 2009). These low copy sequences are more informative for marker discovery, especially for breeding purposes (Courtois et al., 2013). Here, we genotyped 67 accessions of Galapagos tomatoes from the TGRC collection (Supplementary Table S1) using DArTseq and found that *S. cheesmaniae* and *S. galapagense* fall into distinctive clades. Further, we found that the accessions of *S. cheesmaniae* separate based on the island/region from which they originated, and the population structure can be linked to the geological movements of the islands. From this, it can be inferred a clear evolutionary sequence within the Galapagos tomatoes, revealed by molecular, rather than morphological means.

## Materials and Methods

### Plant Material and DNA Isolation

A total of 67 Galapagos tomato accessions – 40 *S. cheesmaniae* and 27 *S. galapagense* -, together with their passport data, were obtained through the Tomato Genetic Resources Center (TGRC) UC, Davis, CA, USA (Supplementary Table S1). In addition, two *S. lycopersicum* varieties (Heinz 1706, and Moneymaker) and one *S. pimpinellifolium* accession (LA0480), also obtained from TGRC, were used for comparison. The one *S. pimpinellifolium* accession was added to compare the Galapagos tomatoes to another wild tomato, while two *S. lycopersicum* varieties were added as references: Heinz 1706 is the variety for which the reference genome sequence was completed (The Tomato Genome Consortium, 2012); and Moneymaker is a popular commercial variety.

To break their dormancy, soften the seed coat, and promote germination, Galapagos seeds were treated with 2.7% sodium hypochlorite solution for 1 h (Rush and Epstein, 1976), then soaked in ddH_2_O for 1 h. Seeds were then placed in magenta boxes with 0.65% agar gel containing 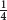 Murashige and Skoog salts for germination. The magenta boxes were kept in a Percival growth chamber at 26°C with a 16 h photoperiod. Seedling tissues, without the root, were harvested when their first true leaf started to emerge. Ten seedlings of the each accession were frozen in liquid nitrogen and ground using sterile mortar and pestle. From this ground tissue, DNA extraction was performed as indicated by DArT Pty Ltd (Canberra, ACT, Australia), in: http://www.diversityarrays.com/sites/default/files/pub/DArT_DNA_isolation.pdf, but with addition of β-mercaptoethanol along with the “fresh buffer.” Washing with CIAA was done three times before addition of isopropanol, and the pellets were dissolved in ddH_2_O. DNA quality and concentration were determined by electrophoresis in 0.8% agarose gel and spectrophotometry using a NanoDrop 2000 (Thermo scientific, Wilmington, DE, USA), and were normalized to a concentration of 100 ng/μL.

### DArTseq Analysis

DArTseq analysis was performed by DArT Pty Ltd. For the purposes of complexity reduction, the gDNA samples were digested with *Pst*I and *Taq*I restriction enzymes. Adapters were ligated to *Pst*I ends and short adapter-ligated fragments were amplified. *PstI*-RE site-specific adapters were tagged with 96 different barcodes to run all DNA samples within a single lane on an Illumina Hiseq2000 (Illumina Inc., San Diego, CA, USA). *PstI* adapters included a sequencing primer site. Quality control was performed by filtering FASTQ files using 90% confidence limits for at least 50% of the bases and further filtering for barcode sequences. The filtered data was then split using a barcode-splitting script and the barcode was trimmed. After trimming the barcode, the average read length was 66 bp with a minimum length of 38 bp and maximum length of 70 bp. The sequences were aligned against a reference constructed by DArT Pty Ltd, from GBS data gathered from the same species, independent of the availability of the whole genome sequence. The short sequence tags were also aligned against the publicly available tomato genome (The Tomato Genome Consortium, 2012) using Bowtie software (Langmead et al., 2009). All alignments were processed using an analytical pipeline developed by DArT Pty Ltd to produce “silicoDArT” tables and “SNP” tables (Cruz et al., 2013). SNP markers were scored 0/1 or 1/0 (homozygous) or 1/1 (heterozygous, scoring the presence of both alleles). The data are available in **Supplementary Data Sheet S1**.

### SNP Filtering

PLINK (Purcell et al., 2007) was used to filter out SNPs with more than 20% missing values and those that were actually or nearly monomorphic (i.e., with minor allele frequencies below 2.5%). Linkage disequilibrium (LD)-based SNP pruning was also performed with PLINK (Purcell et al., 2007), using a pairwise approach with a threshold of *r*^2^ = 0.8.

### Population Structure

The smartPCA application included in the EIGENSOFT 6.0 package (Price et al., 2006) was used to perform a PCA of the SNP dataset that was pruned based on LD. The outlier removal option was disabled.

STRUCTURE software (Pritchard et al., 2000) was run from command line using the admixture model, a burn-in period length of 500,000 and 250,000 MCMC iterations after burn-in. Five independent runs were performed for each *K* from *K* = 1 to *K* = 8. The best number of *K* was then chosen with the DeltaK method (Evanno et al., 2005) by running the Structure Harvester software (Earl and vonHoldt, 2012). Inferred clusters were processed with CLUMPP software (Jakobsson and Rosenberg, 2007) to align the multiple replicate analyses of the same data set and obtain a consensus matrix. Lastly, the DISTRUCT software (Rosenberg, 2004) was used to make a barplot of the Q matrix. The accessions were sorted by island and longitude in the barplot.

An analysis of molecular variance (AMOVA; Excoffier et al., 1992) of the SNP data was done using R package “poppr” (Kamvar et al., 2014) on the dataset of all Galapagos tomato accessions, as well as on *S. cheesmaniae* and *S. galapagense* datasets individually. Significance of the AMOVA and Phi statistic analyses was tested using 999 permutations with the R package “ade4” (Chessel et al., 2004).

### Genetic Distance Tree

The genetic distance matrix was generated from the allelic data (3,974 diploid loci/SNPs) using the simple matching method as a dissimilarity index (Sokal and Michener, 1958), with pair-wise allele deletion when missing data was 40% or higher. The simple matching dissimilarity index was calculated as:

$$d_{\text{i}j}=1-\frac{1}{L}\overset{L}{\underset{l=1}{\sum }}\frac{m_{l}}{\text{π}}$$

where *d_ij_* is the dissimilarity between units *i* and *j, L* is the number of loci, *π* is the ploidy*, m*_l_ is the number of matching alleles for locus *l.*

From the distance matrix, an unweighted Neighbor-Joining tree (Saitou and Nei, 1987) was constructed using the Darwin 6.0 software (Perrier and Jacquemoud-Collet, 2006); branches were tested with 1,000 bootstraps. The tree root is the node of degree 2 in hierarchy.

## Results

### Genotyping by Sequencing and SNP Markers Discovery

**FIGURE 2:**
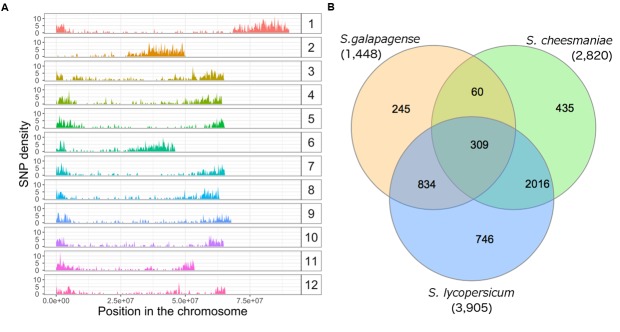
**Single nucleotide polymorphism density and distribution across the 12 chromosomes of the tomato genome and across species. (A)** SNP density across the 12 chromosomes of the tomato genome, obtained after aligning 3,974 SNP markers found in 67 accessions of Galapagos tomatoes, two *S. lycopersicum* varieties and *one S. pimpinellifolium* accession with DArTseq, plotted with ggplot2 package in R. The *x*-axis represents the SNP position along each chromosome (bp). The *y*-axis shows SNP density over the range of the SNP position. Note high SNP density at the edges of the chromosomes. **(B)** Venn diagram of unique and shared SNPs, kept in each dataset after SNP filtering. In this case, the minor allele frequency test was excluded for *S. lycopersicum* due to the small sample of lines available in this study and their low allele diversity. The diagram was drawn from the SNP lists of each species using the InteractiVenn website (Heberle et al., 2015).

We genotyped 67 Galapagos tomato accessions using the DArTseq service from DArT Pty Ltd. A total of 4,887 SNPs were identified in the sequenced fragments. After aligning to the tomato reference genome (The Tomato Genome Consortium, 2012), 282 SNPs aligned to more than two positions. Given that these could be suggestive of repetitive regions, or paralogous sequences, these SNPs were excluded. SNPs that aligned to different locations were considered to be individual SNPs (even if reported as one SNP by DArT). SNP filtering for SNPs with less than 20% missing values and with minor allele frequencies below 2.5%, resulted in 3,974 polymorphic SNP markers left for analysis. Based on their alignment to the tomato reference genome (The Tomato Genome Consortium, 2012), these markers are well distributed across the chromosomes (Supplementary Table S2), with median, mean, and standard deviation of distance between adjacent markers of 91, 338, and 844 kb, respectively (**Figure 2A**). Furthermore, when the data were separated for the individual species and the same parameters were used for SNP filtering, 2,820 SNPs were obtained for *S. cheesmaniae*, 1,448 SNPs for *S. galapagense*, and 3,905 SNPs for *S. lycopersicum*. In the case of *S. lycopersicum*, the minor allele frequency test was excluded due the low allele diversity between the two lines. SNP lists of all species were compared in a Venn diagram (**Figure 2B**), and found that *S. cheesmaniae* shares 71.5% of the SNPs with *S. lycopersicum*, while *S. galapagense* only shares 57.6% of the SNPs with *S. lycopersicum*. From the 2,820 SNPs kept for S. cheesmaniae, the 1,448 SNPs kept for *S. galapagense*, and the 3,905 kept for *S. lycopersicum*, only 360 SNPs are shared among all species and 60 SNPs are shared between the Galapagos tomatoes.

### Germplasm Collection Is Largely Homozygous

We used the SNP markers to estimate the conserved homozygosity in each genotype. The proportion of homozygous markers in each genotype ranges from 96.1 to 99.4% in *S. cheesmaniae* and from 98.2 to 99.4% in *S. galapagense*. The high level of homozygosity could be caused by their propagation at the TGRC. It is also consistent with the early reports by Rick (Rick, 1983), where he described the Galapagos tomatoes to be highly autogamous, due to their flower structure being adapted to self-pollination and claimed that natural populations exist in a virtually pure-line condition. Darwin (2009) also reported low levels of heterozygosity present in the wild specimens of Galapagos tomatoes collected during her expedition to the islands. This strict autogamy, has led to rapid fixation of alleles and accumulation of mutant genes, which contributed largely to the differentiation between populations (Rick, 1983). Moreover, this germplasm collection could be used directly for further genetic studies without the need to develop inbred lines.

### *S. cheesmaniae* and *S. galapagense* Can Be Clearly Differentiated by Genetic Analysis

To dissect the pattern of genetic variation among the accessions, we used both PCA from EIGENSOFT 6.0 (Price et al., 2006) and Bayesian clustering from STRUCTURE software (Pritchard et al., 2000). For PCA, the SNP data was further pruned based on LD, to obtain a subset of SNPs that are in approximate linkage equilibrium with each other. PLINK (Purcell et al., 2007) was used to calculate LD, based on pairwise genotypic correlation with a threshold of *r*^2^= 0.8. LD-based pruning reduced the SNP marker collection to 2,428 SNPs.

**FIGURE 3:**
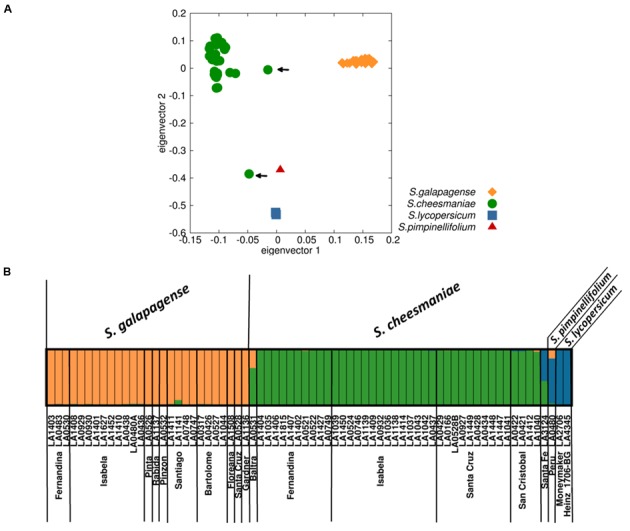
**Population structure of 67 Galapagos tomato accessions based on SNP markers. (A)** Principal component analysis (PCA) of SNP markers identified from 67 samples of *S. galapagense, S. cheesmaniae, S. pimpinellifolium*, and *S. lycopersicum*, where each species/taxon clusters together, except for two accessions (indicated by arrows). **(B)** STRUCTURE analysis with *K* = 3, each accession is represented by a single column, with the color indicating cluster membership.

The PCA showed a clear division between the accessions identified as *S. galapagense* and *S. cheesmaniae*, as well as from *S. lycopersicum* and *S. pimpinellifolium* (**Figure 3A**). The accessions belonging to *S. galapagense* cluster closely together (orange diamonds), while *S. cheesmaniae* accessions are also clustered (green circles), with the exception of two accessions: LA0531 and LA3124 (marked by an arrow), which could be admixtures. Individual PCAs of the accessions belonging to each of *S. galapagense* and *S. cheesmaniae* can be found in the Supplementary Materials (Supplementary Figure S2).

The groups obtained from the PCA were identical with those formed by an alternative clustering program, STRUCTURE (Pritchard et al., 2000), which uses a Bayesian clustering approach to identify the number of populations (K) with the highest structure (Supplementary Figure S3). This is determined by plotting Delta K, based on the method of Evanno et al. (2005); our DeltaK plot showed a peak at *K* = 3 (Supplementary Figure S4), suggesting the presence of three genetically distinct groups that differentiate the two Galapagos tomato species and the tomatoes native to mainland South America (**Figure 3B**). Once more, accessions LA0531 and LA3124 appear to be genetic admixtures. The admix nature of these two accessions was confirmed by the ancestry membership coefficients (Q), which show that LA0531 belongs to both cluster 1, formed by the rest of *S. cheesmaniae* accessions (*Q* = 0.674), and to cluster 2, formed by all *S. galapagense* accessions (*Q* = 0.325), whereas, LA3124 appears to be part of cluster 1 (*Q* = 0.432) and cluster 3, formed by *S. lycopersicum* and *S. pimpinellifolium* (*Q* = 0.568) (Supplementary Table S3). The collection notes of these two accessions, obtained from TGRC database^1^ (Supplementary Table S4), report morphological differences from the typical *S. cheesmaniae* since the time of their collection (Supplementary Figure S1), which confirm that they are hybrids and crossing did not occur during later seed propagation, but in the natural environment. It is also worth noting, that the two accessions are unique to Baltra and Santa Fe, respectively, two very small islands – only one accession was collected from each island (Supplementary Table S1).

**FIGURE 4:**
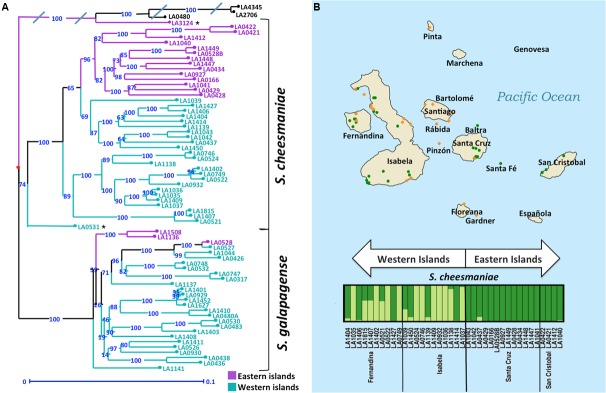
**Clustering of Galapagos tomatoes could be similar to the age of island formation. (A)** Unweighted Neighbor Joining dendrogram, demonstrating genetic distances among 67 accessions of Galapagos tomatoes. Bootstrap = 1,000. Average ‘edge’ distance between bootstrapped trees is 0.1418, 5-percentile: 0.0746, 95-percentile: 0.209. The branches are colored purple for the accessions collected in the eastern islands and turquoise for the accessions collected in the western islands. **(B)** Geographical and genetic distribution of the two groups of Galapagos tomatoes. The orange diamonds represent the collection sites of *S. galapagense*, while the green circles represent the collection sites of *S. cheesmaniae* accessions. STRUCTURE analysis was performed in individual to detect substructure. *S. galapagense* showed no apparent substructure. For *S. cheesmaniae*, *K* = 2 was used. The plot was arranged by island and longitude.

The results from PCA and STRUCTURE are largely consistent. For both analyses we found that *S. galapagense*, *S. cheesmaniae, S. lycopersicum*, and *S. pimpinellifolium* accessions are clearly separated. Although the sampling size of *S. lycopersicum* and *S. pimpinellifolium* accessions is small, the three reference sequences clustered together in all of the analyses performed. Thus, they provide useful reference points to facilitate estimation of the genetic distance between the Galapagos tomato populations. For this purpose, a genetic distance matrix was generated using the simple matching method as a dissimilarity index (Sokal and Michener, 1958). From the distance matrix, an unweighted neighbor-joining tree (Saitou and Nei, 1987) was constructed using Darwin 6.0 software (Perrier and Jacquemoud-Collet, 2006). The neighbor-joining tree revealed a clear differentiation between *S. cheesmaniae* and *S. galapagense* with a bootstrap support of 100% (**Figure 4A**).

### Clustering of *S. cheesmaniae* Accessions Could Be Similar to the Age of Island Formation

In the genetic distance tree, the *S. cheesmaniae* accessions separate into two sub-clusters with a 100% bootstrap support, while the admixtures remained separated from the main *S. cheesmaniae* branches (**Figure 4A**). Likewise, the population structure inferences using STRUCTURE (**Figure 4B**) of *S. cheesmaniae* accessions show two sub-clusters (*K* = 2) and no further structure within the *S. galapagense* group (*K* = 1) (Supplementary Figure S5). *S. galapagense* showed no structure, even when using the No admixture model in STRUCTURE (Pritchard et al., 2000; data not shown).

Interestingly, the division of the *S. cheesmaniae* cluster shows clear geographic structure. The grouping of the accessions matches their region of origin: the accessions collected in the western islands separate clearly from those collected in the eastern islands. This can be linked to the island formation timeline, as all the islands originated at the volcanic hotspot and then moved east with the Nazca plate which holds the Galapagos archipelago (Geist et al., 2014). The eastern islands are therefore older than the western islands (Geist et al., 2014) and from this we can infer that those populations found in the eastern islands may be older than the ones found in the western islands; alternatively, they could be ancestors of the accessions in the west. This inference is supported by the comparison of Wright’s fixation index (F_ST_) values (Wright, 1951) for each *S. cheesmaniae* cluster, obtained from the STRUCTURE analysis (Pritchard et al., 2000). The mean *F*_ST_-value of the accessions from the western islands (0.6239), is considerably higher than the mean *F*_ST_-value of the accessions from the eastern islands (0.2790), which suggests the occurrence of a strong episode of genetic drift on those populations from the newer islands (Falush et al., 2003).

### Analysis of Genetic Variation in Galapagos Tomatoes

An AMOVA (Excoffier et al., 1992) was performed to examine patterns of genetic variation and to estimate variance components at the levels of species and accessions. AMOVA showed that 43.1% of the total variation in the Galapagos tomato populations was explained by differences between the two species; whereas 51% was explained by differences between accessions within the species (**Table 1**). This confirms that the two species are considerably different, but also there is great variation among the accessions within a species.

**Table 1:** Analysis of Molecular Variance (AMOVA) and Monte-Carlo significance tests for the collection of 67 Galapagos tomatoes accession, the group of 40 *S. cheesmaniae* accessions and the group of 27 *S. galapagense* accessions.

	Df	Sum Sq	Mean Sq	Variance %	Sigma	Phi	*P*-value
**Galapagos tomatoes**							
Between species	1	10619	10619	43.1	162	0.43	0.001
Between accessions within species	63	25468	404	51.0	191	0.90	0.001
Within accessions	65	1439	22.1	5.90	22.1	0.94	0.001
Total	129	37526	291	100	375	–	–
***S. cheesmaniae***							
Between region	1	2806	2806	24.3	67.9	0.24	0.001
Between accessions within region	36	14534	404	68.9	192	0.91	0.001
Within accessions	38	718	18.9	6.76	18.9	0.93	0.001
Total	75	18058	241	100	279	–	–
***S. galapagense***							
Between region	1	452	452	5.25	9.99	0.05	0.142
Between accessions within region	25	8649	346	87.1	166	0.92	0.001
Within accessions	27	394	14.6	7.67	14.6	0.92	0.001
Total	53	9495	179	100	190	–	–

*Df, degrees of freedom; Sum Sq, sum of squares; Mean Sq, mean of squares.*

With the purpose of investigating if the region of origin of each accession (east or west of the Galapagos archipelago) had any influence on the genetic variation within the species, further analysis of variance was performed using the region of origin as a factor for each species. These AMOVA revealed that while the most genetic variation occurs between accessions (68.9% in *S. cheesmaniae* and 87.1% in *S. galapagense*), there is a significant variation (24.3%) between regions of origin of the *S. cheesmaniae* accessions (east and west).

## Discussion

We selected SNP markers to measure genetic variation, since SNPs are one of the most common types of genetic variation. Also, they are co-dominant markers, which allow us to estimate the homozygosity of the Galapagos tomatoes germplasm, and thus, their usefulness for genetic studies. The flower morphology of the Galapagos tomatoes suggested their autogamous nature and a high occurrence of inbreeding (Rick and Fobes, 1975). Rick and Fobes (1975) used an allozyme analysis (allelic determined variants of isozymes) to confirm that the variation between populations was greater than the variation within each population. This is consistent with our study, where the lowest percentage of homozygous SNPs was 96.1%. Furthermore, the AMOVA of the allelic data showed significant amount of genetic variance between species and also a significant amount of genetic variance between populations belonging to the same species.

Based on their morphology, *S. cheesmaniae* and *S. galapagense* can be clearly differentiated into two taxonomic groups (**Figure 1**), but results from genetic studies have been contradictory. Rick and Fobes (1975) showed that there was little variation within 54 analyzed accessions, while Darwin (2009) consistently differentiated between the taxonomic groups when analyzing 26 accessions. Nuez et al. (2004) used AFLP analysis on 16 accessions and showed clear differentiation between the taxa (although both taxa were still considered as one species). Lucatti et al. (2013) used 3.3 kb SNP arrays on 34 accessions, but could not differentiate between the two taxa, suggesting they were likely to be morphotypes, rather than two species. However, the SNP array they used was based on sequence information from *S. lycopersicum* (Víquez-Zamora et al., 2013). With current genomic technology, the GBS approach used in this work, DArTseq, enabled a robust genetic characterization of the wild Galapagos tomatoes. By using three different clustering methods – PCA, STRUCTURE and neighbor joining by genetic distance/dissimilarity – we clearly show that *S. cheesmaniae* and *S. galapagense* are two genetically distinct species (**Figure 3**).

In addition, we show that two *S. cheesmaniae* accessions from two very small islands, Baltra and Santa Fe, are admixtures, based on the calculated ancestry membership coefficients (Q) for clustering by STRUCTURE (Supplementary Table S4). One accession (LA3124) appears to be a hybrid with *S. lycopersicum* or *S. pimpinellifolium*; and another accession (LA0531) may be a hybrid with *S. galapagense* (Supplementary Figure S1). Their differences in morphology from the typical *S. cheesmaniae* are reported in the collection notes (Supplementary Table S4). Accession LA0531 comes from two different specimens collected and archived together, whose different morphologies were attributed to depauperation. This may have allowed introgression between *S. cheesmaniae* and *S. galapagense.* Accession LA3124 had big seeds, similar to those of red cherry tomato. Interestingly, LA3124 was previously classified as *S. pimpinellifolium* by Zuriaga et al. (2009), even though the passport data classified it as *S. cheesmaniae.* This highlights its closeness and possible admixture with *S. pimpinellifolium* or *S. lycopersicum*.

*Solanum galapagense* accessions are tightly clustered, indicating little genetic diversity within this species. These results are consistent with Koenig et al. (2013), who suggested the occurrence of strong genetic bottlenecks in *S. galapagense* during island colonization and recent adaptation. If *S. galapagense* populations were established from a small number of individuals that colonized the islands, genetic diversity has not recovered since the founder event. In addition, the isolation of the islands makes the genetic diversity susceptible to genetic drift (Maki, 1999).

In contrast, *S. cheesmaniae* accessions showed clear differentiation between accessions that were collected in the western islands and those collected in the eastern islands. The clustering by region of origin revealed that those accessions in close genetic proximity are also close in geographical origin (**Figure 4**), which suggests a correlation between biodiversity and geography in the Galapagos archipelago. This is consistent with previous reports on biogeography of other endemic species from the Galapagos Islands, such as, marine iguanas, Darwin’s finches, and giant tortoises, among others (Parent et al., 2008). The major factors influencing biogeography in the Galapagos Islands are the volcanic activity and plate tectonics (Merlen, 2014). The study of these, suggests a colonization from east to west (Merlen, 2014), from older to younger islands, supported also by the fact that ocean currents entering the islands from different directions, average an east-west direction (Merlen, 2014). The main islands originated at the volcanic hotspot west of the archipelago and slowly moved east, at approximately 59 km per million years, with the tectonic movement of the Nazca plate (Geist et al., 2014). Newly formed islands at the hotspot are inhospitable, but as they move east, cool down, and erode, the arrival and establishment of life forms becomes possible (Merlen, 2014). At the same time, the subduction of the Nazca plate beneath the South American plate, has caused the oldest islands to drown (Christie et al., 1992), forcing migration of the biodiversity to newer islands. From this, we infer that the eastward movement of the islands could have influenced the gene flow in *S. cheesmaniae* and their adaptation.

Rick and Fobes (1975) reported increased diversity in the Galapagos tomatoes from the western islands and especially from their western slopes. They attributed this increased diversity to the unusually higher precipitation in that area, as plant species richness in the Neotropics is known to be correlated with annual precipitation (Gentry, 1982). Concordantly, our results show that *S. cheesmaniae* populations in the western islands seem to have higher levels of genetic diversity than those in the eastern islands (**Figure 4B**). This may be because fewer populations of *S. cheesmaniae* exist in the eastern islands, but it could also be attributed to the founder effect of possible colonization events from western islands to eastern islands. East-to-west colonization permits the dispersal of species from the older islands to the newest islands as their volcanic activity decreases, soil develops, and they become habitable (Merlen, 2014). However, there is still some volcanic activity in the older islands that can destroy the island’s flora. Re-colonization after volcanism in the eastern islands, by a few individuals from the western islands, would also reduce genetic diversity in the eastern islands.

The division of *S. cheesmaniae* in two groups, has also been reported by Nuez et al. (2004), based on internode length, *S. cheesmaniae* “short” and *S. cheesmaniae* “long.” However, it is not yet clear if the genetic-geographical clustering coincides with the morphological separation. Given that Nuez et al. (2004) made their own collection and only took samples in the central islands, their data and the results presented here cannot be compared. Further morphological characterization of the TGRC collection could confirm if the whole collection of *S. cheesmaniae* could be separated into “short” and “long” morphotypes and if this division would be consistent with the geographical division.

No substructure was found in the *S. galapagense* collection. This is likely to be due to a relatively recent divergence of this species. Interestingly, there are no reports *of S. galapagense* from any of the eastern islands. The combination of the distribution and lower genetic diversity leads us to hypothesize that *S. galapagense* is a relatively new species that diverged from *S. cheesmaniae* after the eastern islands became isolated.

According to estimates from Geist et al. (2014), the eastern islands of San Cristobal, Santa Fe, and Española emerged approximately 2.4–4.0 million years ago, whereas, the first western islands with the presence of *S. galapagense*, Floreana and Santa Cruz, emerged somewhere around 1.1 and 2.3 million years ago. Geist et al. (2014) also estimated that Floreana and Santa Cruz were in peak volcanic activity two million years ago, making colonization impossible, while the volcanic activity of San Cristobal was on the decline at that time as it drifted away from the hotspot and became detached from Santa Cruz and Floreana. It was only one million years later that volcanic activity in Floreana and Santa Cruz decreased sufficiently for colonization to occur (Geist et al., 2014). Thus, divergence time of *S. galapagense* can be estimated to have occurred roughly one million years ago, when Floreana and Santa Cruz, the last of the eastern islands without *S. galapagense*, became a suitable habitat for life. These estimates are consistent with previous reports by Nesbitt and Tanksley (2002), who suggested that the initial radiation of the genus *Lycopersicon* occurred over seven million years ago, and that *S. lycopersicum* (then referred to as *L. esculentum*) and its closest relatives (which include *S. galapagense* (then referred to as *L. cheesmanii*, accession LA0483) and *S. pimpinellifolium* (then referred to as *L. pimpinellifolium*) diverged from a common ancestor approximately one million years ago. A more recent divergence time (between 0.19 and 0.29 million years ago) was suggested by Strickler et al. (2015) for *S. lycopersicum* and *S. galapagense*. However, the estimates by Strickler et al. (2015) are in reference to the Heinz 1706 variety, which, in the same publication, was found to contain several regions of significant introgressions from *S. pimpinellifolium* (Strickler et al., 2015), which could bias the estimates of species divergence time.

To our knowledge, there is not an estimate of *S. cheesmanie* divergence from a common ancestor. If the colonization of Galapagos Islands was east to west, then *S. cheesmaniae* could be an older species than *S. galapagense*, and could even be an ancestor to it. Rick and Fobes (1975) suggested that *S. cheesmaniae* could possibly be closer to the original stem line of the red-fruited species than any other member. He argued that their autogamous reproduction and the lack of competition in the Galapagos could have resulted in its preservation as an ancient biotype (Rick and Fobes, 1975). However, the lower genetic variation in *S. cheesmaniae* found in the older islands could be due to a founder effect, and colonization could have happened from west to east. If this was the case, *S. cheesmaniae* and *S. galapagense* could have diverged around the same time from the same ancestor.

The recent divergence of the tomato clade species and their close relationship has made their phylogenetic classification difficult, especially with the casual occurrence of interspecific hybridization (Zuriaga et al., 2009). Many attempts to determine the phylogeny have been made using diverse methods (Peralta and Spooner, 2001; Peralta et al., 2005; Spooner et al., 2005; Zuriaga et al., 2009; The 100 Tomato Genome Sequencing Consortium et al., 2014; Strickler et al., 2015; Dodsworth et al., 2016), but the results are not consistent. Most publications agree that there is a close relationship among the red-fruited group, which contains *S. lycopersicum, S. cheesmaniae, S. galapagense*, and *S. pimpinellifolium*. However, they have not yet reached a consensus on the relationship order within the red-fruited group. According to Zuriaga et al. (2009), using only a few accessions of each species in phylogenetic studies could be the cause of conflicting phylogeny results. Adding intraspecific variation to phylogenetic studies could improve the resolution. Our study provides a rich intraspecific dataset, which could be used to further characterize the phylogeny of the tomato clade. Meanwhile, we found a fine phylogenetic relationship between accessions (**Figure 4A**) and significant intraspecific structure in *S. cheesmaniae*, corresponding to the age of the island of origin of each accession (Supplementary Figure S6A). Interestingly, the accessions coming from the eastern islands of Santa Cruz and San Cristobal, are closely related within each island and separate from each other, while the more diverse accessions from the western islands, Fernandina and Isabela, are interrelated across the two islands. Additionally, *S. galapagense* accessions also group by island of origin (Supplementary Figure S6B), further demonstrating the influence of the islands’ biogeography on the Galapagos tomatoes gene flow.

To conclude, we propose a likely sequence of events for the diversification and speciation of wild tomatoes on the Galapagos Islands which is not only of evolutionary interest, in the classic evolutionary “laboratory” of the Galapagos Islands, but which also provides guidance for the strategic discovery of diversity, such as of novel stress tolerance alleles (Rush and Epstein, 1976; Firdaus et al., 2013), useful for future improvement of cultivated tomato, the largest horticultural crop globally.

## Author Contributions

YP, SS, SN, and MT designed research. YP and SS performed research. IP and SH contributed analytic tools. YP, SS, and MT discussed data analysis. YP analyzed data. YP, SS, and MT wrote the paper.

## Conflict of Interest Statement

The authors declare that the research was conducted in the absence of any commercial or financial relationships that could be construed as a potential conflict of interest.

## Footnotes

1 http://tgrc.ucdavis.edu/

## Supplementary Material

DATA SHEET S1 **SNP data for Galapagos tomatoes.** The table shown in the ‘SNPs’ worksheet contains all SNP data, obtained from DArTseq analysis, for 40 *S. cheesmaniae* accessions, 27 *S. galapagense* accessions, one *S. pimpinellifolium* accession and two *S. lycopersicum* varieties. The SNP markers initially scored 0/1 or 1/0 (homozygous) or 1/1 (heterozygous, scoring the presence of both alleles) were translated to the specific nucleotides found at each allele (A = adenine, T = thymine, G = guanine, C = cytosine, 0 = missing data). Each of the SNP markers has a unique identifier number. The data sheet contains 19 columns of information per SNP marker. The description of each column can be found in the same file, in the ‘SNPsMetadataDefinitions’ worksheet. To approximate the position of the SNP marker in the genome, the sequenced reads were aligned to the tomato genome version 9 downloaded from ftp://ftp.jgi-psf.org/pub/compgen/phytozome/v9.0/Slycopersicum/assembly/Slycopersicum_225.fa.gz. Aligner: blastn, E-value: 5E-05, Min. base identity: 80%.
