# Supplementary material for: Genetic Diversity and Population Structure of Two Tomato Species from the Galapagos Islands
Source: Front Plant Sci. 2017 Feb 15;8:138. doi: 10.3389/fpls.2017.00138 (PMC5309213; doi:10.3389/fpls.2017.00138)
Supplement: Supplementary file 2 [file Table_1.DOCX]

**Supplementary Table S1. List of Galapagos tomato accessions and their collection details.** Source: (TGRC, <http://tgrc.ucdavis.edu/>)

| **Accession** | **Species** | **Collection site** | **Collection year** | **Latitude** | **Longitude** |
| --- | --- | --- | --- | --- | --- |
| LA0166 | *S. cheesmaniae* | Santa Cruz: Barranco, N of Punta Ayora | 1950 | -0.750 | -90.317 |
| LA0317 | *S. galapagense* | Bartolome | 1954 | -0.283 | -90.550 |
| LA0421 | *S. cheesmaniae* | San Cristobal: cliff East of Wreck Bay | 1956 | -0.898 | -89.609 |
| LA0422 | *S. cheesmaniae* | San Cristobal: Wreck Bay, Puerto Baquerizo | 1956 | -0.895 | -89.610 |
| LA0426 | *S. galapagense* | Bartolome: E of landing | 1956 | -0.283 | -90.550 |
| LA0428 | *S. cheesmaniae* | Santa Cruz: Trail Bellavista to Miconia Zone | 1956 | -0.650 | -90.300 |
| LA0429 | *S. cheesmaniae* | Santa Cruz: Crater in highlands | 1956 | -0.644 | -90.329 |
| LA0434 | *S. cheesmaniae* | Santa Cruz: Rambech Trail | 1956 | -0.733 | -90.300 |
| LA0436 | *S. galapagense* | Isabela: Villamil | 1956 | -0.953 | -90.978 |
| LA0437 | *S. cheesmaniae* | Isabela: Ponds North of Villamil | 1956 | -0.953 | -90.978 |
| LA0438 | *S. galapagense* | Isabela: coast at Villamil | 1956 | -0.978 | -91.021 |
| LA0480A | *S. galapagense* | Isabela: Cowley Bay | 1957 | -0.400 | -90.983 |
| LA0483 | *S. galapagense* | Fernandina: inside crater | 1956 | -0.367 | -91.550 |
| LA0521 | *S. cheesmaniae* | Fernandina: Inside Crater | 1957 | -0.367 | -91.550 |
| LA0522 | *S. cheesmaniae* | Fernandina: Outer slopes | 1957 | -0.367 | -91.550 |
| LA0524 | *S. cheesmaniae* | Isabela: Punta Essex | 1957 | -1.000 | -91.433 |
| LA0526 | *S. galapagense* | Pinta: W Side | 1957 | 0.583 | -90.783 |
| LA0528 | *S. galapagense* | Santa Cruz: Academy Bay | 1957 | -0.750 | -90.317 |
| LA0528B | *S. cheesmaniae* | Santa Cruz: Academy Bay | 1957 | -0.750 | -90.317 |
| LA0530 | *S. galapagense* | Fernandina: crater | 1957 | -0.367 | -91.550 |
| LA0531 | *S. cheesmaniae* | Baltra: Barranco slope, N side | 1958 | -0.417 | -90.283 |
| LA0532 | *S. galapagense* | Pinzon: NW side | 1958 | -0.600 | -90.683 |
| LA0746 | *S. cheesmaniae* | Isabela: Punta Essex | 1960 | -1.000 | -91.417 |
| LA0747 | *S. galapagense* | Santiago: Cape Trenton | 1960 | -0.367 | -90.583 |
| LA0748 | *S. galapagense* | Santiago: E Trenton Islet | 1960 | -0.370 | -90.584 |
| LA0749 | *S. cheesmaniae* | Fernandina: North side | 1960 | -0.263 | -91.472 |
| LA0927 | *S. cheesmaniae* | Santa Cruz: Academy Bay | 1964 | -0.742 | -90.308 |
| LA0929 | *S. galapagense* | Isabela: Punta Flores | 1961 | -0.100 | -91.467 |
| LA0930 | *S. galapagense* | Isabela: Punta Tortuga | 1962 | -0.215 | -91.391 |
| LA0932 | *S. cheesmaniae* | Isabela: Tagus Cove | 1964 | -0.273 | -91.366 |
| LA1035 | *S. cheesmaniae* | Fernandina: Low elevation | 1964 | -0.429 | -91.583 |
| LA1036 | *S. cheesmaniae* | Isabela: far north end | 1964 | -0.133 | -91.350 |
| LA1037 | *S. cheesmaniae* | Isabela: Alcedo crater | 1965 | -0.423 | -91.117 |
| LA1039 | *S. cheesmaniae* | Isabela: Cape Berkeley | 1965 | -0.039 | -91.533 |
| LA1040 | *S. cheesmaniae* | San Cristobal: Caleta Tortuga | 1965 | -0.726 | -89.384 |
| LA1041 | *S. cheesmaniae* | Santa Cruz: El Cascajo | 1965 | -0.669 | -90.265 |
| LA1042 | *S. cheesmaniae* | Isabela: Cerro Santo Tomas | 1965 | -0.921 | -90.994 |
| LA1043 | *S. cheesmaniae* | Isabela: Cerro Santo Tomas | 1966 | -0.887 | -91.004 |
| LA1044 | *S. galapagense* | Bartolome | 1966 | -0.284 | -90.548 |
| LA1136 | *S. galapagense* | Gardner-near-Floreana Islet | 1967 | -1.333 | -90.295 |
| LA1137 | *S. galapagense* | Rabida: N side | 1967 | -0.400 | -90.710 |
| LA1138 | *S. cheesmaniae* | Isabela: E of Cerro Azul | 1968 | -0.883 | -91.267 |
| LA1139 | *S. cheesmaniae* | Isabela: W of Cerro Azul | 1968 | -0.933 | -91.417 |
| LA1141 | *S. galapagense* | Santiago: N crater | 1968 | -0.200 | -90.750 |
| LA1401 | *S. galapagense* | Isabela: N of Punta Tortuga | 1971 | -0.237 | -91.391 |
| LA1402 | *S. cheesmaniae* | Fernandina: W of Punta Espinoza | 1971 | -0.294 | -91.552 |
| LA1403 | *S. galapagense* | Fernandina: W of Punta Espinoza | 1971 | -0.294 | -91.552 |
| LA1404 | *S. cheesmaniae* | Fernandina: W flank caldera | 1970 | -0.367 | -91.617 |
| LA1406 | *S. cheesmaniae* | Fernandina: SW rim caldera | 1970 | -0.350 | -91.567 |
| LA1407 | *S. cheesmaniae* | Fernandina: caldera, NW bench | 1970 | -0.351 | -91.556 |
| LA1408 | *S. galapagense* | Isabela: SW volcano, Cape Berkeley | 1970 | -0.046 | -91.559 |
| LA1409 | *S. cheesmaniae* | Isabela: Punta Albemarle | 1971 | -0.133 | -91.367 |
| LA1410 | *S. galapagense* | Isabela: Punta Ecuador | 1971 | -0.347 | -91.051 |
| LA1411 | *S. galapagense* | Santiago: N James Bay | 1971 | -0.203 | -90.829 |
| LA1412 | *S. cheesmaniae* | San Cristobal: opposite Isla Lobos | 1971 | -0.867 | -89.567 |
| LA1414 | *S. cheesmaniae* | Isabela: Cerro Azul | 1971 | -0.983 | -91.217 |
| LA1427 | *S. cheesmaniae* | Fernandina: WSW rim of caldera | 1971 | -0.383 | -91.550 |
| LA1447 | *S. cheesmaniae* | Santa Cruz: Charles Darwin Station-Punta Nunez | 1971 | -0.733 | -90.283 |
| LA1448 | *S. cheesmaniae* | Santa Cruz: Puerto Ayora, Pelican Bay | 1971 | -0.733 | -90.300 |
| LA1450 | *S. cheesmaniae* | Isabela: Bahia San Pedro | 1971 | -0.743 | -90.305 |
| LA1452 | *S. galapagense* | Isabela: E slope, Volcan Alcedo | 1969 | -0.974 | -91.440 |
| LA1508 | *S. galapagense* | Floreana: Corona del Diablo Islet | 1972 | -0.383 | -91.067 |
| LA1627 | *S. galapagense* | Isabela: Darwin's Lake | 1974 | -1.216 | -90.423 |
| LA1815 | *S. cheesmaniae* | Fernandina: NW rim caldera | 1970 | -0.250 | -91.367 |
| LA3124 | *S. cheesmaniae* | Santa Fe: near E landing | 1991 | -0.350 | -91.567 |
