# Supplementary material for: Genetic Diversity and Population Structure of Two Tomato Species from the Galapagos Islands
Source: Front Plant Sci. 2017 Feb 15;8:138. doi: 10.3389/fpls.2017.00138 (PMC5309213; doi:10.3389/fpls.2017.00138)
Supplement: Supplementary file 3 [file Table_2.DOCX]

**Supplementary Table S2. SNP distribution in chromosomes after SNP filtering** (<20% missing values, minor allele frequencies below 2.5% and LD pruned with pairwise method r^2^=0.8)

| Chromosome | 0 | 1 | 2 | 3 | 4 | 5 | 6 | 7 | 8 | 9 | 10 | | 11 | 12 |
| --- | --- | --- | --- | --- | --- | --- | --- | --- | --- | --- | --- | --- | --- | --- |
| Number of SNPs | 205 | 545 | 371 | 364 | 325 | 245 | 331 | 264 | 268 | 273 | 276 | | 271 | 236 |
| Number of SNPs (LD pruned) | 49 | 354 | 234 | 237 | 208 | 161 | 227 | 170 | 164 | 162 | 146 | 173 | | 143 |
