# Supplementary material for: Genetic Diversity and Population Structure of Two Tomato Species from the Galapagos Islands
Source: Front Plant Sci. 2017 Feb 15;8:138. doi: 10.3389/fpls.2017.00138 (PMC5309213; doi:10.3389/fpls.2017.00138)
Supplement: Supplementary file 4 [file Table_3.docx]

**Supplementary Table S3. Q-matrix of STRUCTURE analysis.** Estimated ancestry membership coefficients (Q).

|  | **Inferred clusters**  3 | | |
| --- | --- | --- | --- |
| **Accession** | 1 | 2 | 3 |
| LA0166 | 0.996 | 0.004 | 0.000 |
| LA0317 | 0.001 | 0.996 | 0.004 |
| LA0421 | 0.985 | 0.000 | 0.015 |
| LA0422 | 0.984 | 0.000 | 0.015 |
| LA0426 | 0.004 | 0.996 | 0.000 |
| LA0428 | 0.998 | 0.000 | 0.001 |
| LA0429 | 1.000 | 0.000 | 0.000 |
| LA0434 | 1.000 | 0.000 | 0.000 |
| LA0436 | 0.002 | 0.993 | 0.005 |
| LA0437 | 0.989 | 0.001 | 0.010 |
| LA0438 | 0.005 | 0.995 | 0.000 |
| LA0480A | 0.008 | 0.991 | 0.001 |
| LA0483 | 0.000 | 0.995 | 0.005 |
| LA0521 | 0.990 | 0.010 | 0.000 |
| LA0522 | 0.999 | 0.001 | 0.000 |
| LA0524 | 1.000 | 0.000 | 0.000 |
| LA0526 | 0.000 | 0.999 | 0.000 |
| LA0528 | 0.000 | 1.000 | 0.000 |
| LA0528B | 1.000 | 0.000 | 0.000 |
| LA0530 | 0.000 | 1.000 | 0.000 |
| LA0531 | 0.674 | 0.325 | 0.000 |
| LA0532 | 0.000 | 1.000 | 0.000 |
| LA0746 | 1.000 | 0.000 | 0.000 |
| LA0747 | 0.000 | 1.000 | 0.000 |
| LA0748 | 0.000 | 1.000 | 0.000 |
| LA0749 | 1.000 | 0.000 | 0.000 |
| LA0927 | 1.000 | 0.000 | 0.000 |
| LA0929 | 0.000 | 1.000 | 0.000 |
| LA0930 | 0.005 | 0.995 | 0.000 |
| LA0932 | 0.999 | 0.001 | 0.000 |
| LA1035 | 1.000 | 0.000 | 0.000 |
| LA1036 | 1.000 | 0.000 | 0.000 |
| LA1037 | 0.999 | 0.001 | 0.000 |
| LA1039 | 1.000 | 0.000 | 0.000 |
| LA1040 | 0.975 | 0.025 | 0.000 |
| LA1041 | 1.000 | 0.000 | 0.000 |
| LA1042 | 1.000 | 0.000 | 0.000 |
| LA1043 | 1.000 | 0.000 | 0.000 |
| LA1044 | 0.000 | 1.000 | 0.000 |
| LA1136 | 0.000 | 1.000 | 0.000 |
| LA1137 | 0.000 | 1.000 | 0.000 |
| LA1138 | 1.000 | 0.000 | 0.000 |
| LA1139 | 1.000 | 0.000 | 0.000 |
| LA1141 | 0.080 | 0.920 | 0.000 |
| LA1401 | 0.000 | 1.000 | 0.000 |
| LA1402 | 1.000 | 0.000 | 0.000 |
| LA1403 | 0.001 | 0.999 | 0.000 |
| LA1404 | 1.000 | 0.000 | 0.000 |
| LA1406 | 1.000 | 0.000 | 0.000 |
| LA1407 | 1.000 | 0.000 | 0.000 |
| LA1408 | 0.008 | 0.992 | 0.000 |
| LA1409 | 1.000 | 0.000 | 0.000 |
| LA1410 | 0.000 | 1.000 | 0.000 |
| LA1411 | 0.007 | 0.993 | 0.000 |
| LA1412 | 1.000 | 0.000 | 0.000 |
| LA1414 | 1.000 | 0.000 | 0.000 |
| LA1427 | 1.000 | 0.000 | 0.000 |
| LA1447 | 1.000 | 0.000 | 0.000 |
| LA1448 | 1.000 | 0.000 | 0.000 |
| LA1449 | 1.000 | 0.000 | 0.000 |
| LA1450 | 1.000 | 0.000 | 0.000 |
| LA1452 | 0.000 | 1.000 | 0.000 |
| LA1508 | 0.001 | 0.999 | 0.000 |
| LA1627 | 0.002 | 0.998 | 0.000 |
| LA1815 | 1.000 | 0.000 | 0.000 |
| Moneymaker | 0.003 | 0.001 | 0.996 |
| LA3124 | 0.432 | 0.000 | 0.568 |
| Heinz 1706 | 0.000 | 0.000 | 1.000 |
| LA0480  *(S. pimpinellifolium*) | 0.022 | 0.148 | 0.831 |
| LA0527 | 0.000 | 1.000 | 0.000 |
