# Supplementary material for: Genetic Diversity and Population Structure of Two Tomato Species from the Galapagos Islands
Source: Front Plant Sci. 2017 Feb 15;8:138. doi: 10.3389/fpls.2017.00138 (PMC5309213; doi:10.3389/fpls.2017.00138)
Supplement: Supplementary file 5 [file Table_4.DOCX]

**Supplementary Table S4: Collection notes for admixes.** Source: (TGRC, <http://tgrc.ucdavis.edu/>)

| **Accession** | **Summary of collection notes** |
| --- | --- |
| LA0531 | Two different specimens (SAL259 and SAL260) were collected, approximately 20 meters apart, in the North of Baltra by Miguel Castro in 1958. SAL259 was initially identified as a *S. pimpinellifolium* or a typical *S. cheesmaniae*, the leaves were simple with round outline, slight serration, long internodes and slightly hairy, the sepals were short, and the fruits were small and jointed; while SAL260 looked similar to SAL259, except for shorter internodes and serrated leaves. These differences were attributed to “probably depauperating effects” and the two specimens were archived as one accession. |
| LA3124 | Three plants of similar morphology were collected in 1991 in Barrington beach, Santa Fe, just above water line. Their morphology was described as with few short hairs, leaf shape of the red cherry (like typical *S.* *cheesmaniae*), small flowers, stigma not exerted, simple inflorescence, yellow fruit, unusually large seeds (dimensions of red cherry) and jointed pedicels |
