# Supplementary figures and images for: Genetic Diversity and Population Structure of Two Tomato Species from the Galapagos Islands
Source: Front Plant Sci. 2017 Feb 15;8:138. doi: 10.3389/fpls.2017.00138 (PMC5309213; doi:10.3389/fpls.2017.00138)

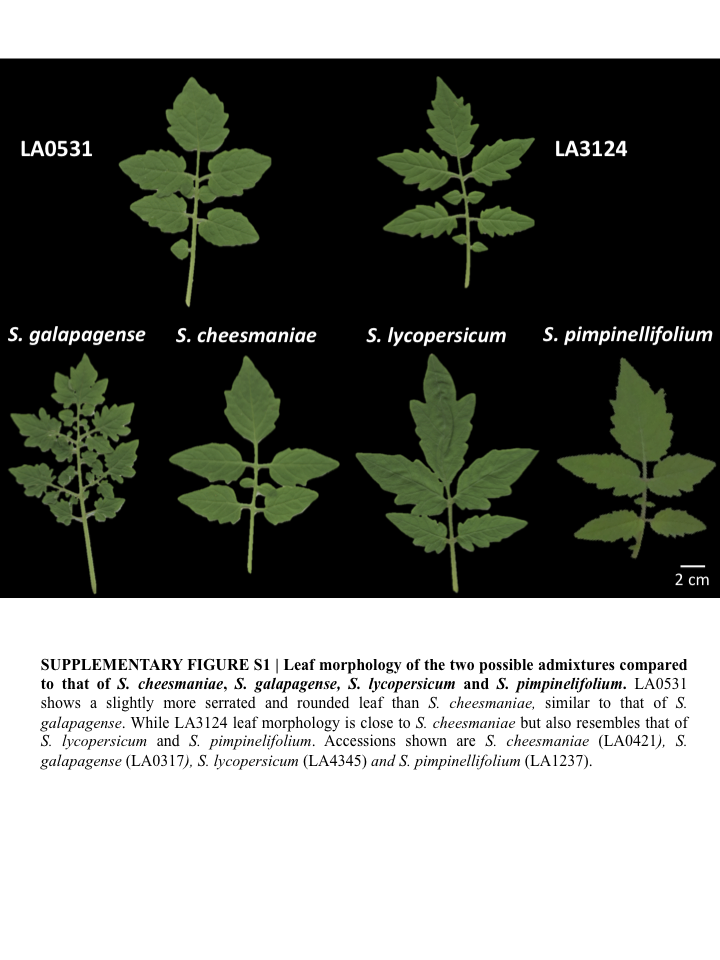

Supplement: Supplementary file 6 [file Image_1.TIFF]

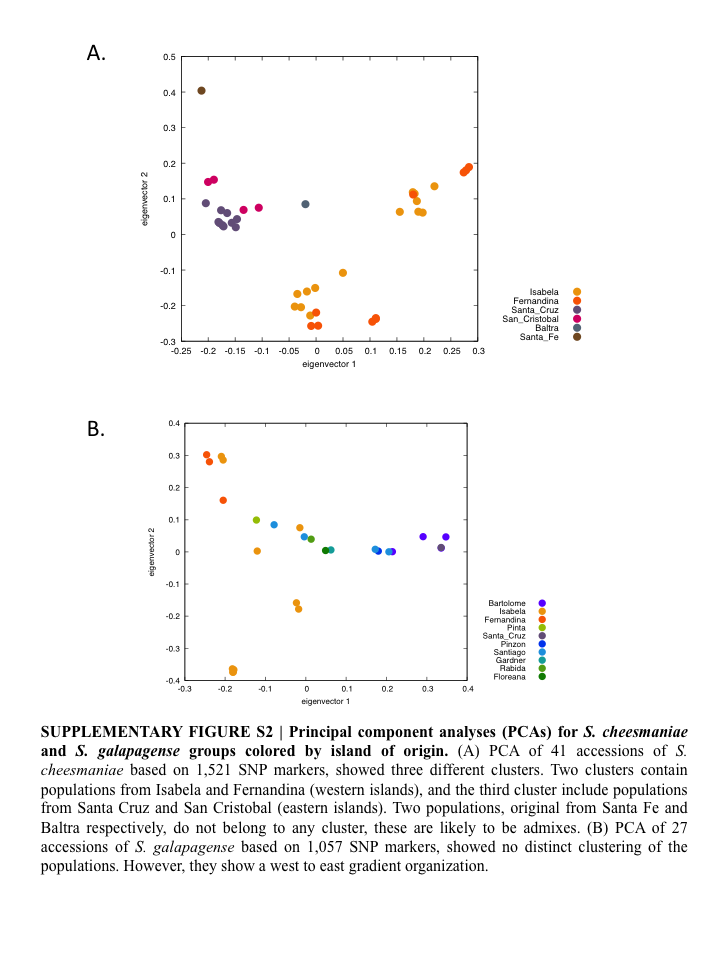

Supplement: Supplementary file 7 [file Image_2.TIFF]

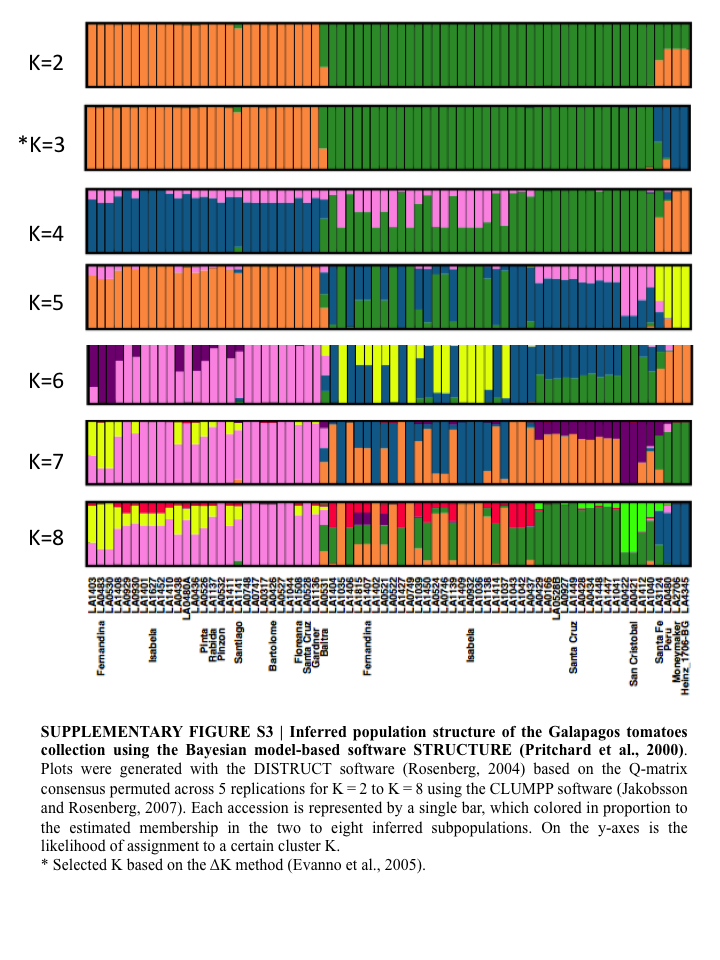

Supplement: Supplementary file 8 [file Image_3.tiff]

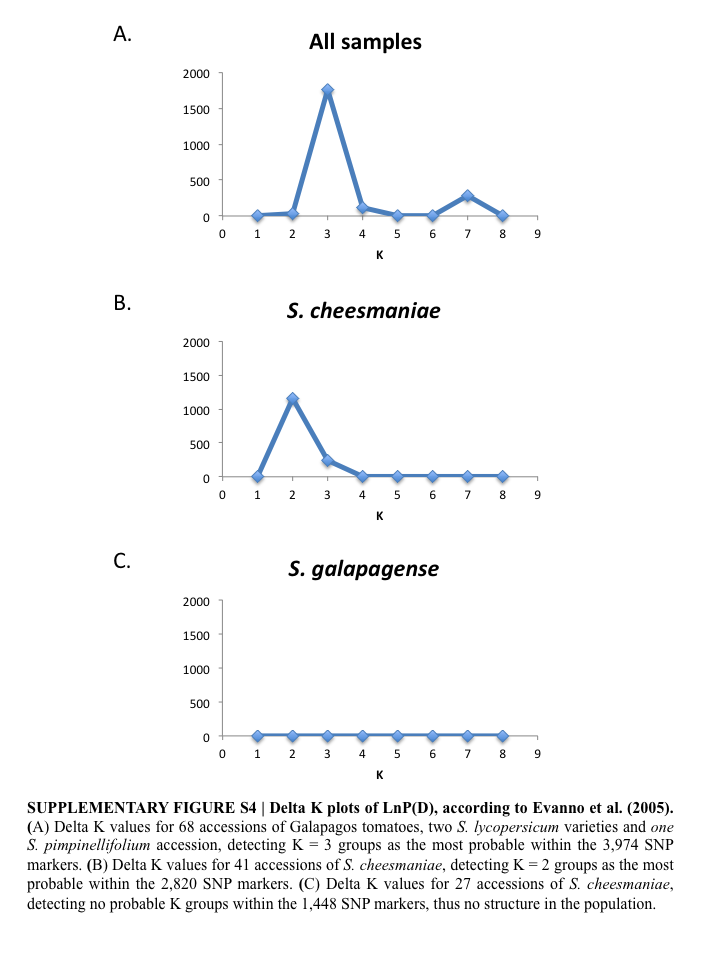

Supplement: Supplementary file 9 [file Image_4.tiff]

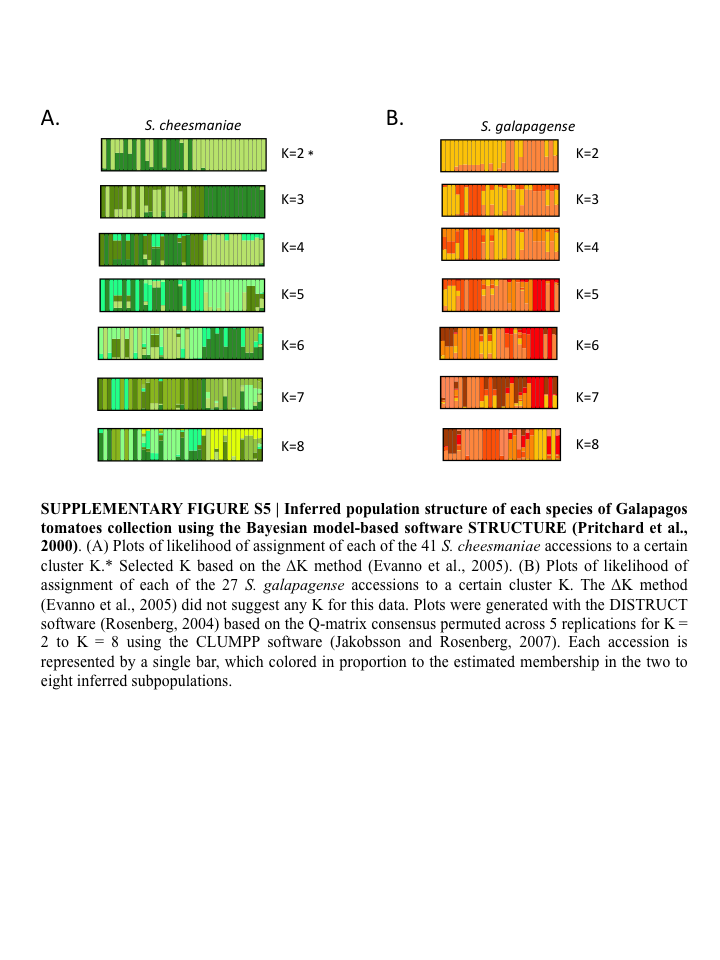

Supplement: Supplementary file 10 [file Image_5.TIFF]

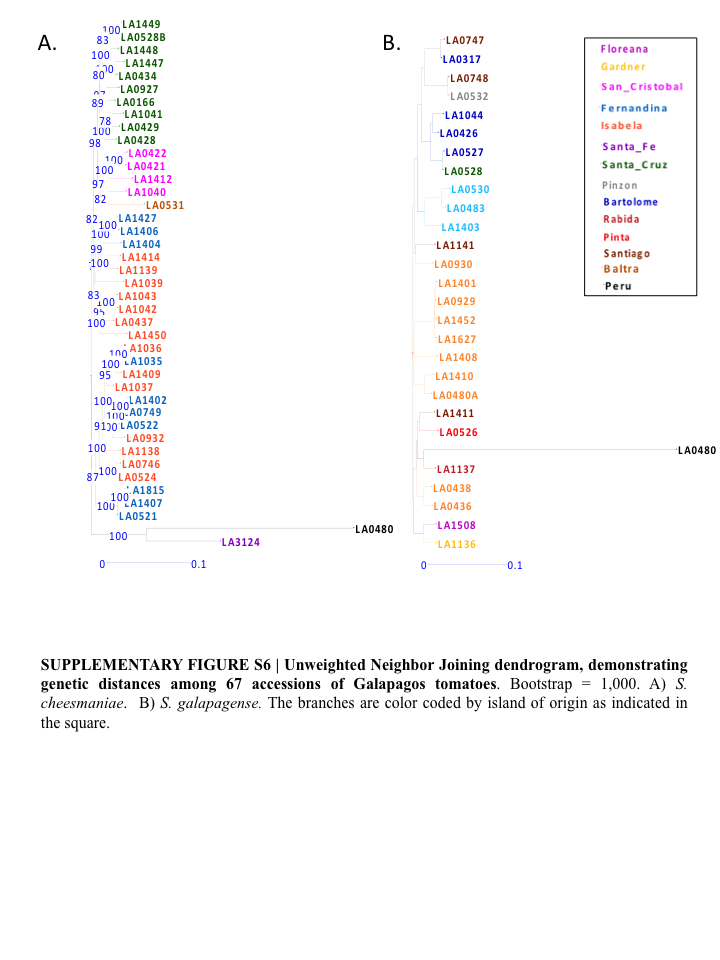

Supplement: Supplementary file 11 [file Image_6.TIFF]
